# Cytokine signature and COVID-19 prediction models in the two waves of pandemics

**DOI:** 10.1038/s41598-021-00190-0

**Published:** 2021-10-21

**Authors:** Serena Cabaro, Vittoria D’Esposito, Tiziana Di Matola, Silvia Sale, Michele Cennamo, Daniela Terracciano, Valentina Parisi, Francesco Oriente, Giuseppe Portella, Francesco Beguinot, Luigi Atripaldi, Mario Sansone, Pietro Formisano

**Affiliations:** 1 Department of Translational Medicine, Federico II University of Naples, Naples, Italy; 2 URT “Genomic of Diabetes”, Institute of Experimental Endocrinology and Oncology (IEOS), National Research Council (CNR), Naples, Italy; 3 Azienda Ospedaliera Dei Colli, Naples, Italy; 4 Department of Electrical Engineering and Information Technology, Federico II University of Naples, Naples, Italy

**Keywords:** Biomarkers, Infectious diseases

## Abstract

In Europe, multiple waves of infections with SARS-CoV-2 (COVID-19) have been observed. Here, we have investigated whether common patterns of cytokines could be detected in individuals with mild and severe forms of COVID-19 in two pandemic waves, and whether machine learning approach could be useful to identify the best predictors. An increasing trend of multiple cytokines was observed in patients with mild or severe/critical symptoms of COVID-19, compared with healthy volunteers. Linear Discriminant Analysis (LDA) clearly recognized the three groups based on cytokine patterns. Classification and Regression Tree (CART) further indicated that IL-6 discriminated controls and COVID-19 patients, whilst IL-8 defined disease severity. During the second wave of pandemics, a less intense cytokine storm was observed, as compared with the first. IL-6 was the most robust predictor of infection and discriminated moderate COVID-19 patients from healthy controls, regardless of epidemic peak curve. Thus, serum cytokine patterns provide biomarkers useful for COVID-19 diagnosis and prognosis. Further definition of individual cytokines may allow to envision novel therapeutic options and pave the way to set up innovative diagnostic tools.

## Introduction

Coronavirus disease-19 (COVID-19) has been initially defined as an atypical pneumonia caused by a zoonotic viral agent, then identified as Severe Acute Respiratory Syndrome Coronavirus-2 (SARS-CoV-2)^1^.

In the first wave of the outbreak, most people with COVID-19 developed mild disease (40%), without evidence of viral pneumonia or hypoxia, or moderate disease (40%), with clinical signs of pneumonia (fever, cough, dyspnea, fast breathing) but no signs of reduced oxygen saturation (SpO_2_ ≥ 90% on room air). 15–20% of infected individuals developed a severe or critical disease with complications such as respiratory failure, acute respiratory distress syndrome, sepsis and septic shock, thromboembolism, and/or multiorgan failure, including acute kidney injury and cardiac injury. *Exitus* was eventually reported in two to eight weeks from symptom appearance^2^.

Many countries have faced a second wave of COVID-19 pandemics. Compared to the first wave, a lower proportion of patients requiring invasive mechanical ventilation and a lower rate of thrombotic events have been observed^3,4^. Hospitalized patients in the second wave are younger, require fewer days of hospitalization, have longer survival^5,6^.

Although COVID-19 is mostly defined by pneumonia, it has been documented that extrapulmonary systemic hyperinflammation plays a crucial role in clinical manifestations^7^, also contributing to COVID-19 associated coagulopathy^8^. Peculiar COVID-19 immunophenotypes have been also described^7,9^. At peripheral blood level, a decreased number of basophils and plasmacytoid dendritic cell depletion correlates with disease severity^9^. Aberrant pathogenic T cells and inflammatory monocytes are rapidly activated and produce a large number of cytokines, thus inducing a so called “cytokine storm”. Many studies on first wave of COVID-19 outbreak have indicated an increase of both pro-inflammatory and anti-inflammatory cytokines, whose levels appear to correlate with severity of disease, both in adults and in children^9–12^. Hence, targeted approaches have been envisioned to dampen COVID-related cytokine storm, particularly IL-6, IL-8, and TNFα^13–15^. However, to date, no available cytokine-based drug or therapy have demonstrated 100% efficacy for patients with COVID-19.

Since March 2020, a large number of studies on cytokine storm in COVID-19 patients has been published. Main findings often display a high degree of variability and refer to the first wave of the outbreak^11^. In this study, we have provided a cytokine profile of patients with mild and severe symptoms of COVID-19 during two peaks of epidemic curves in Campania region (Italy). Moreover, by using machine learning methods, we have analyzed whether a specific cytokine profile could guide disease diagnosis and prognosis.

## Methods

### Study design and ethics statement

Between March 2020 and May 2020, 65 consecutive patients with a positive SARS-CoV-2 PCR swab test, admitted at Federico II University Hospital and “Azienda Ospedaliera dei Colli” Hospital of Naples, Italy, were recruited for the study. 49 healthy adult volunteers were also enrolled as control cohort.

Similarly, during the second wave of pandemics, from September to October 2020, 36 patients with confirmed SARS-CoV-2 infection and 15 negative controls were included in the study.

The study was approved by the ethical committee of the University of Naples Federico II (prot. n. 140/20/ESCOVID19). All the methods involving patients and volunteers have been performed in accordance with the Declaration of Helsinki. Also, an informed consent has been obtained from all participants.

### Sample processing and cytokine assay

Blood samples in serum separator tubes were centrifuged and stored at − 80 °C. Serum samples were then screened for the concentration of Interleukin (IL)-1β, IL-1ra, IL-2, IL-4, IL-5, IL-6, IL-7, IL-8, IL-9, IL-10, IL-12(p70), IL-13, IL-15, IL-17, basic Fibroblast Growth Factor (FGF-b), Eotaxin, Granulocyte-Colony Stimulating Factor (G-CSF), Granulocyte–Macrophage Colony Stimulating Factor (GM-CSF), Interferon (IFN)-γ, Interferon gamma-Induced Protein (IP)-10, Monocyte Chemoattractant Protein (MCP)-1, Macrophage Inflammatory Protein 1-alpha/beta (MIP-1α, MIP-1β), Platelet-Derived Growth Factor (PDGF), Regulated on Activation Normal T-cell Expressed and Secreted RANTES/CCL5, Tumor Necrosis Factor (TNF)-α, and Vascular Endothelial Growth Factor (VEGF), using the Bio-Plex multiplex Human Cytokine and Growth factor kits (Bio-Rad) according to the manufacturer's protocol and as previously described^16^.

### Statistical analysis

A Shapiro–Wilk test was used to evaluate whether the continuous data were normal distributed, and according to the results, values were expressed as median and interquartile range and compared using the Kruskall-Wallis non-parametric test followed by Mann Whitney U test for pairwise comparisons. The non-parametric Jonckheere–Terpstra test was used to analyse trend between an ordinal independent variable. Categorical values were described by number of occurrences and percentages and compared by chi-square test.

Three machine learning methods have been used for prediction of COVID-19: linear discriminant analysis (LDA), classification and regression tree (CART) and neural network (NNET).Performance of algorithms in terms of sensitivity, specificity and overall accuracy were computed.

The predictive accuracy of the single factors and of the machine learning methods was measured by the area under the receiver operating characteristic (ROC) curve (AUC)^17^.

Algorithms have been first designed (trained) and then evaluated (test) on proper sets of data. To avoid overfitting and to robustly evaluate classification performance, a cross-validation approach was used. In detail, one of the subjects was excluded from the training set and used as test: the procedure was iterated over all the subjects and average performance were thus computed. Thisleave-one-out approach better suites for small data-sets^18,19^:

Data from the first wave of outbreak have been used to produce cross-validated classifiers (LDA, CART): those classifiers have been then applied on data from the second wave. Performance have been evaluated using confusion matrix indices (sensitivity, specificity, overall accuracy).

Processing and statistical analysis have been conducted using R software. Differences were considered statistically significant for *p* value less than 0.05.

## Results

### Wave 1 cytokine signature

Between March 2020 and May 2020, 65 patients with a positive SARS-CoV-2 PCR test were enrolled. In agreement with World Health Organization (WHO) eight-point scale for COVID-19 trial endpoints^20^, patients were classified in “mild” (WHO scores 3–4; N = 46) and “severe” (WHO scores 5–8; N = 19). A cohort of 49 healthy blood donors was enrolled as control. No differences for gender were observed in the three groups (Table 1). Severe COVID-19 patients were significantly older compared with both mild COVID-19 patients and controls. At variance, no differences in age were detected between mild COVID-19 patients and controls (Table 1).

**Table 1:** *Serum concentration (pg/ml) of cytokines, chemokines and growth factors (Wave 1).* Results are expressed as median and range [25% percentile; 75% percentile] or number of cases (%). Jonckheere–Terpstra test was used to assess the trend between groups. The non parametric Kruskall Wallis test was applied to assess the difference among three groups followed by Mann Whitney U test for pairwise comparisons.

	CONTROL (n = 49)	MILD COVID-19 (n = 46)	SEVERE COVID-19 (n = 19)	Trend *p* value	Overall *p* value	Mild versus control	Severe versus control	Severe versus mild
Gender, male n. (%)	24 (48.9)	27 (58.7)	15 (78.9)		0.0795			
Age	53 [49; 56]	59 [45; 68]	67 [60; 76]		0.0085		0.0066	
IL-1β	3.55 [3.32; 4.34]	4.82 [3.96; 5.71]	4.82 [3.96; 7.59]	< 0.0001	< 0.0001	< 0.0001	0.0065	
IL-1ra	252 [194.1; 394.6]	371 [284.1; 723.5]	889.6 [417.1; 2060]	< 0.0001	< 0.0001	0.0002	< 0.0001	0.0477
IL-2	11.98 [11.33; 12.96]	13.41 [12.93; 14.67]	14.85 [12.93; 16.79]	< 0.0001	< 0.0001	0.0001	< 0.0001	
IL-4	2.04 [1.73; 2.47]	3.08 [2.64; 3.65]	3.22 [2.35; 4.35]	< 0.0001	< 0.0001	< 0.0001	< 0.0001	
IL-5	67.89 [62.28; 76.56]	99.43 [86.37; 109.5]	105.6 [84.07; 136.7]	< 0.0001	< 0.0001	< 0.0001	< 0.0001	
IL-6	5.56 [4.83; 6.07]	11.3 [8.07; 26.98]	92.39 [37.95; 157.3]	< 0.0001	< 0.0001	< 0.0001	< 0.0001	0.0170
IL-7	28.5 [26.44; 30.76]	38.66 [33.48; 44.79]	37.28 [28.24; 46.15]	< 0.0001	< 0.0001	< 0.0001	0.0003	
IL-8	16.09 [13.42; 18.6]	69.71 [32.63; 176.7]	171.5 [63.86; 1122]	< 0.0001	< 0.0001	< 0.0001	< 0.0001	
IL-9	301.8 [280.3; 319.2]	294.4 [272.5; 333.4]	280.5 [193.9; 314]	0.23	0.3484			
IL-10	7.39 [6.69; 8.01]	11.1 [9.98; 13.46]	16.19 [12.17; 22.07]	< 0.0001	< 0.0001	< 0.0001	< 0.0001	
IL-12 (p70)	11.26 [10.84; 12.88]	12.31 [11.27; 13.23]	12.84 [11.66; 13.43]	0.005	0.0018	0.0031	0.0398	
IL-13	5.75 [4.81; 6.64]	7.02 [3.91; 12.18]	3.46 [2.83; 9.89]	0.704	0.0780			
IL-15	255.4 [243.5; 278.1]	333.9 [303.2; 364.6]	258.6 [276.6; 405.9]	< 0.0001	< 0.0001	< 0.0001	< 0.0001	
IL-17	22.88 [20.18; 25.48]	26.22 [23.86; 28.61]	27.81 [25.43; 30.61]	< 0.0001	< 0.0001	0.0011	0.0009	
Eotaxin	55.24 [38.54; 70.51]	73.8 [39.91; 114.3]	49.88 [17.39; 78.83]	0.481	0.0328			
FGF-b	65.31 [60.11; 70.38]	71.88 [65.66; 76.76]	69.54 [59.83; 78.74]	0.015	0.0124	0.0093		
G-CSF	111.7 [91.71; 127]	199.5 [155.5; 269.6]	285.4 [200.4; 581.7]	< 0.0001	< 0.0001	< 0.0001	< 0.0001	
GM-CSF	7.72 [7.41; 8.15]	8.79 [7.57; 10.39]	9.27 [7.19; 14.4]	< 0.0001	0.0012	0.0033	0.0183	
IFN-γ	19.43 [18.1; 22.22]	28.29 [23.51; 31.09]	37.02 [27.78; 63.27]	< 0.0001	< 0.0001	< 0.0001	< 0.0001	
IP-10	526.4 [425.1; 636.7]	1490 [780.1; 4387]	3547 [1734; 10989]	< 0.0001	< 0.0001	< 0.0001	< 0.0001	
MCP-1	45.29 [35.99; 67.23]	133.1 [63.27; 185.5]	312 [69.55; 604.6]	< 0.0001	< 0.0001	< 0.0001	< 0.0001	
MIP-1α	2.73 [2.49; 2.97]	6.45 [4.715; 10.17]	15.3 [6.95; 57.51]	< 0.0001	< 0.0001	< 0.0001	< 0.0001	
MIP-1β	376.7 [351.3; 410.4]	363.1 [329.2; 393.7]	382.1 [206.1; 583.1]	0.404	0.3104			
RANTES	34,222 [25916; 42539]	24,109 [15796; 45255]	22,739 [2683; 108506]	0.056	0.1442			
TNF-α	86.85 [81.23; 100.8]	104.9 [90.9; 120.3]	121.5 [73.9; 150.5]	< 0.0001	0.0016	0.0104	0.0077	
VEGF	394.2 [360.4; 422.3]	618.2 [524.2; 710.4]	571.4 [477.2; 814.5]	< 0.0001	< 0.0001	< 0.0001	< 0.0001	
PDGF	1497 [1230; 1933]	3046 [2063; 4705]	1838 [555.4; 5089]	0.001	< 0.0001	< 0.0001		0.0249

**Figure 1:**
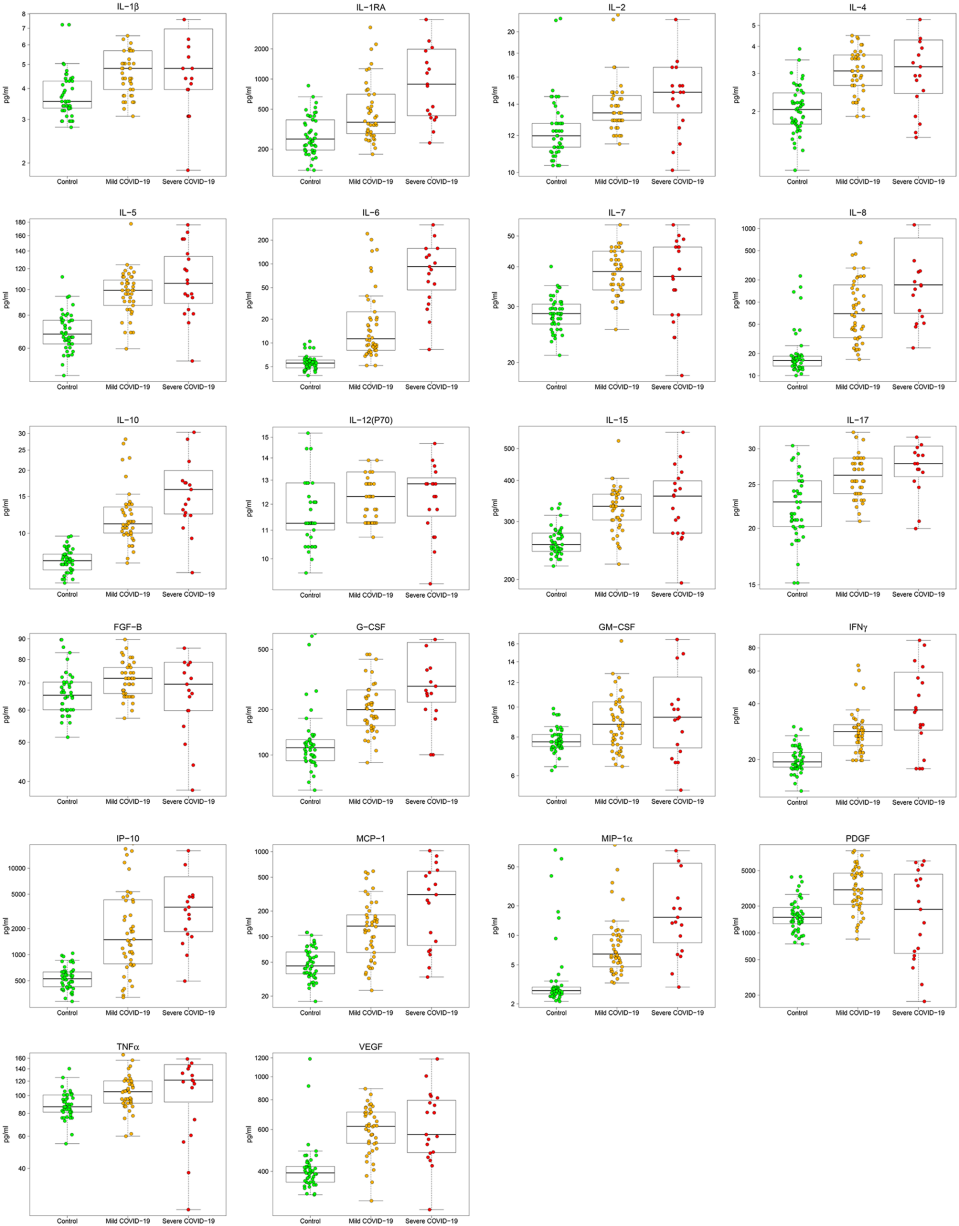
COVID-19 patients display increased trend in circulating cytokines. Box plots denote median and 25th to 75th percentiles (boxes) and minimum to maximum (whiskers) and Jonckheere–Terpstra trend test was performed to analyse data. Figure reports only factors with statistically significant different trends. *p* values and the number of patients for each group are reported in Table 1.

A significant increasing trend of IL-1β, IL-1ra, IL-2, IL-4, IL-5, IL-6, IL-7, IL-8, IL-10, IL-12(p70), IL-15, IL-17, FGF-b, G-CSF, GM-CSF, IFN-γ, IP-10, MCP-1, MIP-1α, PDGF, TNF-α, and VEGF was observed in the three groups (Controls ≤ Mild COVID-19 ≤ Severe COVID-19) (Table 1, Fig. 1). Moreover, significantly higher concentration of all these factors was detected in serum of mild and severe COVID-19 patients compared to controls (Table 1). Only FGF-b did not change between severe COVID-19 and controls (Table 1). Finally, IL-1ra and IL-6 levels were significantly higher in severe versus mild COVID-19 patients, while PDGF decreased (Table 1).

**Figure 2:**
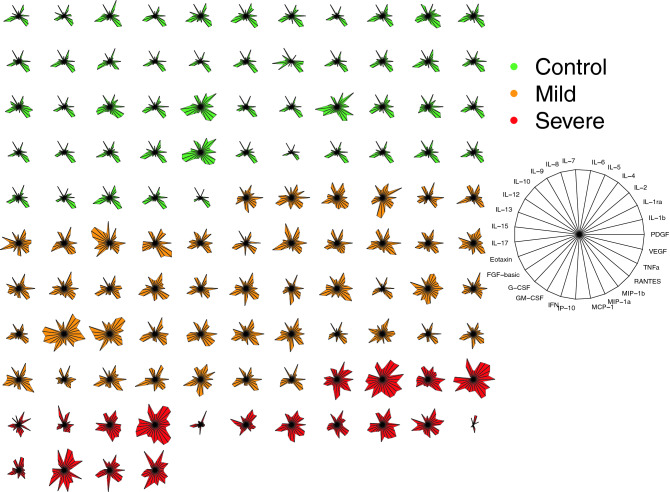
Cytokine-based pattern of COVID-19 patients. Star plot obtained by multivariate data analysis of whole cytokinome of every subject consists of a sequence of equi-angular spokes (radii), with each spoke representing one cytokine as indicated in figure legend on the right. Data length of a spoke is proportional to the magnitude of the variable for the data point relative to the maximum magnitude of the variable across all data points. A line is drawn connecting the data values for each spoke.

Thus, patients with COVID-19 displayed higher levels of cytokines and chemokines, as also shown by the starplot in Fig. 2.

### Cytokine-based prediction models

**Figure 3:**
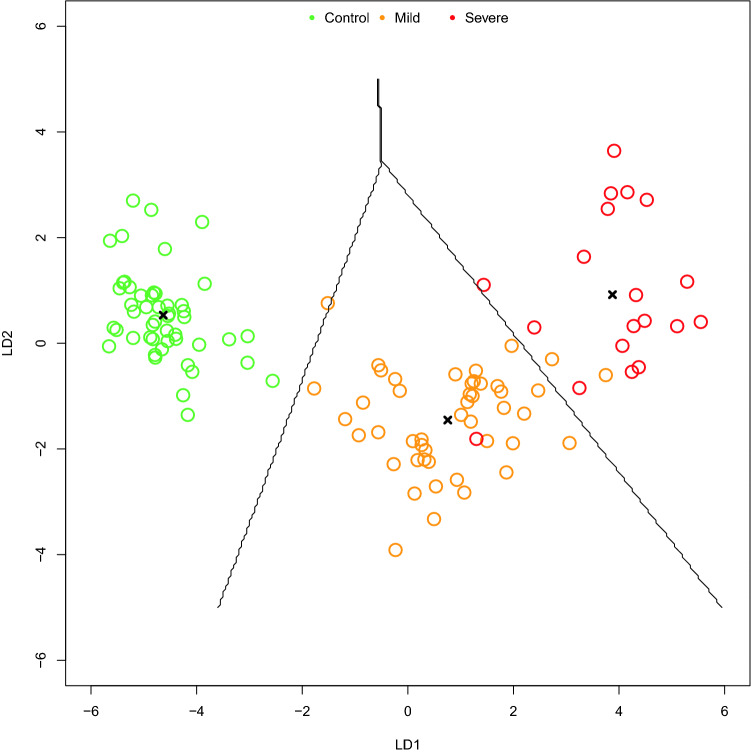
27 cytokine-based algorithm allows to predict disease state and severity. 2D scatterplot of each subject’s cytokines. LDA projection is based on two-component LD1 and LD2 whose coefficients are reported in Supplementary Table 3.

**Figure 4:**
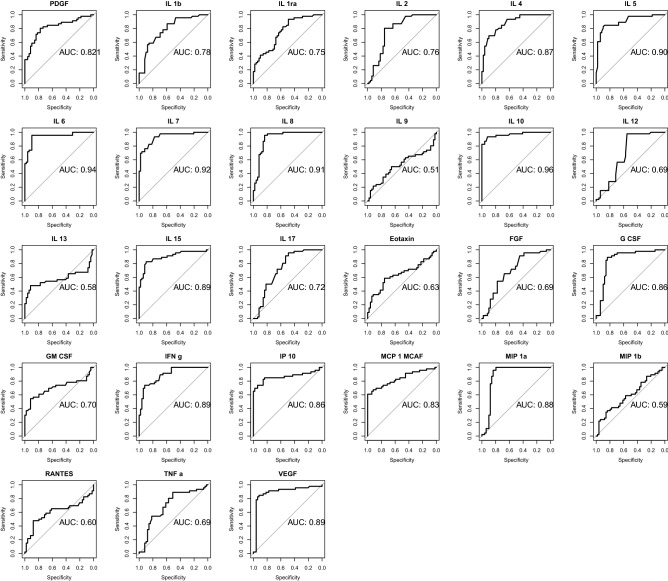
Diagnostic relevance of COVID-19 related cytokines. The diagnostic performance of cytokines, chemokines and growth factors was estimated using ROC curve analysis and compared with the AUC in Controls versus Mild + Severe COVID-19 patients.

**Figure 5:**
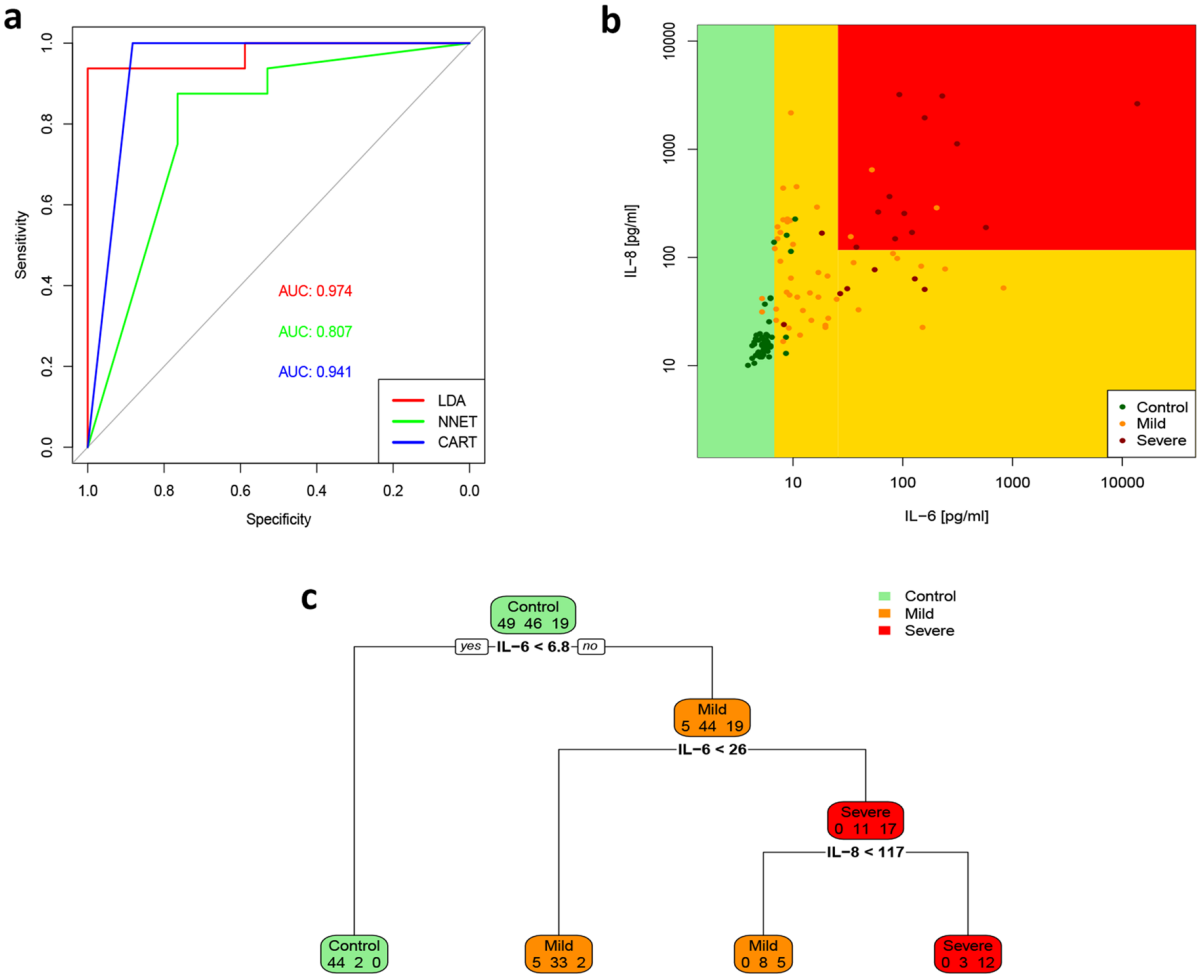
IL-6 and IL-8 performance in discriminating COVID-19 disease and severity. Comparison of classification accuracy of LDA, NNET and CART algorithms. AUC of ROC analysis indicates performance of the three classifier algorithms (**A**). Scatterplot from CART analysis identifies the groups labelled by their terminal nodes (**B**). The decision tree shows the rules and split points to estimate COVID-19 disease and severity. In each box, the first number estimates controls, the second number estimates mild COVID-19 patients, the third number severe COVID-19 patients. Decision binary tree reveals an optimal cut-off of IL-6 > 6.8 pg/ml for predicting COVID-19 disease and of IL-8 > 117 pg/ml for severity (**C**).

Next, we attempted to define a cytokine-based COVID-19 prediction model. As shown in Fig. 3, LDA algorithm allowed to classify subjects in the three groups (control, mild COVID-19, severe COVID-19) with accuracy 0.96, 95% CI: (0.91, 0.99) (Fig. 3, Supplementary Tables 1–3). ROC analyses revealed that almost all cytokines could achieve high diagnostic discriminative power (Fig. 4). However, IL-6, IL-8, IL-10 and IP-10 showed either diagnostic and prognostic classification performance, with an AUC > 0.95 in at least 2 out of the 3 groups (Control vs mild + severe COVID-19; mild vs control + severe COVID-19; severe vs control + mild COVID-19) (Supplementary Table 4). Thus, machine learning algorithms (LDA, NNET and CART) were set up using only the concentrations of the four selected cytokines. ROC analyses revealed a high performance of the three classifier algorithms, with an AUC of 0.97 for LDA, 0.81 for NNET and 0.94 for CART (Fig. 5A). Indeed, CART algorithm clearly indicated that IL-6 discriminated controls and COVID-19 patients. Moreover, combination of IL-6 and IL-8 well defined disease severity. Test overall accuracy was 0.85; 95% CI: (0.77, 0.91) (Fig. 5B,C, Supplementary Tables 5, 6).

### Wave 2 cytokine signature

During September and October 2020 (wave 2), other 36 patients with confirmed SARS-CoV-2 infection and 15 negative controls were enrolled. 26 patients were classified as mild ad 10 patients as severe. As for wave 1, significant differences and increasing trends of IL-1β, IL-1ra, IL-2, IL-6, IL-8, IL-10, GM-CSF, IFN-γ, IP-10, were observed in the three groups (Controls ≤ Mild COVID-19 ≤ Severe COVID-19) (Table 2). At variance, compared to wave 1, neither significant trend, neither significant difference was detected for IL-4, IL-5 IL-7, IL-17, FGF-b, G-CSF, PDGF, TNF-α, and VEGF among the three groups (Table 2). Interestingly, in wave 2, only IL-1ra, IL-2, IL-6 IL-8 and IFN-γ concentrations were significantly higher in serum of mild and severe COVID-19 patients compared to controls (Table 2). Higher concentrations of IL-1β and IL-12(p70) were detected only in serum from mild COVID-19 patients, while IP-10 only in serum of severe COVID-19 patients, compared to controls (Table 2). Reduced levels of eotaxin in mild COVID-19 patients were also observed (Table 2). Notably, compared to wave 1, only IP-10 concentrations were significantly higher in severe versus mild COVID-19 patients, with no change of IL-1ra and IL-6 levels (Table 2).

**Table 2:** *Serum concentration (pg/ml) of cytokines, chemokines and growth factors (Wave 2).* Results are expressed as median and range [25% percentile; 75% percentile] or number of cases (%). Jonckheere–Terpstra test was used to assess the trend between groups. The non parametric Kruskall Wallis test was applied to assess the difference among three groups followed by Mann Whitney U test for pairwise comparisons.

	CONTROL (n = 15)	MILD COVID-19 (n = 26)	SEVERE COVID-19 (n = 10)	Trend *p* value	Overall *p* value	Mild versus control	Severe versus control	Severe versus mild
Gender, male n. (%)	10 (66.6)	20 (76.9)	9 (90)		0.4022			
Age	56 [49; 59]	59 [50; 77]	65 [62; 73]		0.0085		< 0.0001	0.0078
IL-1β	4.28 [4.18; 4.61]	4.93 [4.36; 5.68]	4.71 [4.39; 5.09]	0.035	0.0157	0.0134		
IL-1ra	358.9 [261.7; 549.7]	748.7 [515.3; 1851]	962.3 [439.9; 3550]	0.002	0.0027	0.0036	0.0251	
IL-2	12.46 [11.98; 13.41]	13.89 [12.93; 15.33]	13.89 [13.29; 14.85]	0.004	0.0017	0.0017	0.0360	
IL-4	4.21 [3.36; 5.32]	4.35 [3.65; 5.46]	3.51 [3.22; 4.64]	0.604	0.3044			
IL-5	93.28 [81.01; 102.5]	98.66 [90.2; 108.7]	91.74 [89.43; 109.1]	0.514	0.4529			
IL-6	6.31 [5.18; 7.03]	11.19 [9.18; 14.51]	22.84 [9.52; 78.53]	< 0.0001	< 0.0001	< 0.0001	< 0.0001	
IL-7	44.79 [38.66; 52.86]	48.17 [43.27; 60.34]	42.76 [37.97; 56.48]	0.943	0.3576			
IL-8	22.13 [18.09; 25.65]	41.24 [31.73; 60.87]	62.63 [38.2; 90.45]	< 0.0001	0.0001	0.0014	0.0003	
IL-9	235.8 [218.4; 257	233.4 [195.7; 261.4]	237.7 [195.2; 248.7]	0.427	0.6491			
IL-10	9.08 [8.33; 9.84]	11.58 [10.13; 14.36]	17.02 [11.68; 28.74]	< 0.0001	< 0.0001	0.0018	< 0.0001	
IL-12 (p70)	11.53 [11.01; 11.79]	12.31 [11.79; 12.97]	12.05 [11.27; 12.97]	0.056	0.0202	0.0157		
IL-13	3.04 [2.83; 3.25]	3.57 [3.19; 5.15]	3.14 [2.83; 3.3]	0.154	0.0017	0.0019		
IL-15	265.7 [231.6; 287.4]	336.4 [282; 384.9]	291.3 [268.4; 322.5]	0.052	0.0014	0.0009		
IL-17	28.61 [27.81; 34.26]	30.61 [26.22; 35.08]	29.41 [27.61; 33.95]	0.844	0.8307			
Eotaxin	205.9 [130.2; 293.6]	134.1 [104.3; 270.4]	100.9 [78.22; 153.6]	0.004	0.0171		0.0140	
FGF-b	85.36 [78.74; 91.78]	85.36 [76.48; 98.54]	84.27 [75.33; 96.49]	0.694	0.8380			
G-CSF	166.4 [131.4; 207.2]	189.3 [162.2; 226.1]	190.2 [165.2; 445.5]	0.038	0.1057			
GM-CSF	7.38 [6.81; 7.57]	9.45 [7.85; 10.3]	8.04 [7.38; 8.55]	0.041	0.0009	0.0006		
IFN-γ	24.76 [21.78; 28.79]	32.37 [28.92; 43.05]	36.24 [30.07; 64.68]	< 0.0001	0.0008	0.0044	0.0024	
IP-10	1081 [722.6; 1382]	1932 [1187; 5275]	8769 [4991; 21726]	< 0.0001	< 0.0001		< 0.0001	0.0083
MCP-1	65.46 [48.44; 99.57]	103.1 [73.89; 179.2]	160.9 [61.1; 364]	0.019	0.0531			
MIP-1α	3.56 [3.12; 8.05]	6.34 [4.28; 8.22]	8.06 [3.96; 21.83]	0.016	0.0530			
MIP-1β	271.5 [246.9; 300.1]	289.2 [266.6; 327.3]	288.3 [255; 318.9]	0.215	0.2513			
RANTES	20,323 [14757; 27267]	25,675 [14405; 35015]	23,107 [15863; 34575]	0.377	0.5413			
TNF-α	118.4 [108; 132.6]	131 [100.5; 137]	128.3 [109.2; 160.1]	0.253	0.5333			
VEGF	425.3 [380.3; 622]	603.2 [468.4; 681.9]	491.9 [403.7; 524.2]	0.498	0.0867			
PDGF	3593 [2289; 6284]	6240 [3989; 9332]	4052 [2542; 12542]	0.121	0.0652			

### Challenge of cytokine-based prediction model

To validate cytokine-based COVID-19 prediction algorithms defined with data of the first wave (Fig. 5), CART analysis was carried out using data of the second wave as test sample. Test accuracy was 0.68; 95% CI: (0.54, 0.80), with a low sensitivity for the discrimination of severe COVID-19 and low specificity for mild COVID-19 patients (Supplementary Tables 7, 8).

Thus, a prediction model was set up with cytokine data derived only from control and mild COVID-19 cohorts of wave 1. ROC analyses revealed that IL-5, IL-6, IL-7, IL-8 and IL-10 showed the best discriminative power (Supplementary Table 9). Based on these selected cytokines, CART algorithm indicated that IL-6 was able to discriminate control and mild COVID-19 patients with an overall test accuracy of 0.92; 95% CI: (0.85, 0.97) (Supplementary Tables 10, 11). Interestingly, challenge of this prediction model with data from the second wave achieved an accuracy of 0.83; 95% CI: (0.68, 0.93), sensitivity of 0.88 and specificity of 0.73 (Supplementary Tables 12, 13), indicating IL-6 as the best predictor of COVID-19.

## Discussion

COVID-19, caused by the SARS-CoV-2, leads to fast activation of innate immune cells, with a profound cytokine response, especially in patients developing severe disease, resembling a hyper- inflammatory state^21^. The identification of specific cytokines as indicators of disease severity might improve clinical management of COVID-19 patients having a great impact on the diagnostic and therapeutic decision making. However, discrepancies exist on factors involved in cytokine storm and the majority of studies refers only to the first wave of outbreak.

Here, we have shown that: (1) second wave of COVID-19 pandemics is characterized by a less impressive cytokine storm compared to wave 1; (2) 27 cytokine-based algorithm allows to predict disease state and severity with an accuracy of about 96%; (3) IL-6 was significantly associated with COVID-19 diagnosis regardless of peak epidemic curve.

Accumulating evidence has clearly indicated that cytokine storm occurs in patients with COVID-19; however, the different cytokine profiles analyzed revealed variable results. Consistent with previous studies^12,22^, results obtained in our population during the wave 1 reveal an activation of type 1, type 2 and type 3 immunity. In detail, we found increased levels of many pro-inflammatory and suppressive cytokines, as well as chemokines and growth factors, including IL-1β, IL-1ra, IL-2, IL-4, IL-5, IL-6, IL-7, IL-8, IL-10, IL-12(p70), IL-15, IL-17, FGF-b, G-CSF, GM-CSF, IFN-γ, IP-10, MCP-1, MIP-1α, PDGF, TNF-α, and VEGF. IL-6 and IL-1ra levels further increased in patients who were critically ill. It is widely recognized that IL-6, an important biomarker of inflammation for multiple conditions, has a crucial role in COVID-19 cytokine storm^23,24^. Its levels correlate with serum viral load detected by RT-PCR in critically ill COVID-19 patients and with disease outcome^23–25^. IL-1ra has inhibitory roles against pro-inflammatory cytokine activation and T lymphocyte responses^26,27^. It regulates IL-1, TNF-α and IFN production^27^, arguing a potential role in constraining a further increase of these cytokines in severe patients. The simultaneous increase of IL-6 and IL-1ra in critically ill patients suggests an overactive immune response, which may participate to the inflammation-induced tissue damage.

Cytokines display a large interindividual variability, and their functions and release depend on multiple signals, different cell targets, physiological and lifestyle factors. Thus, it is particularly challenging to evaluate cytokines’ diagnostic ability due to the difficulty of setting up cytokine cut-off levels^28,29^. Notably, here we have shown that a 27-cytokine profiling could be used to stratify patients with COVID-19. However, currently, a diagnostic tool based on the measurement of the whole cytokinome may raise problems for high costs to the National Health Systems.

Thus, we selected IL-6, IL-8, IL-10 and IP-10 as the cytokines with the highest performance in the discrimination of mild COVID-19, severe COVID-19 patients and healthy volunteers. Our results are in agreement with the work by Laing and colleagues who identified IL-6, IL-10 and IP-10 as a “severity-related triad”^9^. IL-10 is a cytokine with anti-inflammatory functions. It suppresses macrophage and dendritic cell activation and limits Th1 and Th2 effector responses^28^. In COVID-19, IL-10 could be involved in counteracting the hyperactive immune response, thereby limiting injury but also boosting infection persistence. IP-10 has versatile biological functions on different cell types, which include chemoattraction of inflammatory cells, but also migration and proliferation of endothelial cells^30^. IP-10 is commonly secreted in response to IFNγ. However, it could be directly induced also by virus-related mechanisms^9^. IL-8 is a potent pro-inflammatory cytokine. It is involved in the recruitment and activation of neutrophils^31,32^. Thus, its increase may be related to the neutrophilia often detected in patients with COVID-19.

Interestingly, these four cytokines were fed into three machine learning methods: CART, NNET, and LDA. We found that all these methods were able to predict COVID-19 occurrence and severity with a comparable high performance. Although LDA and NNET provided superior or comparable accuracy, CART is considered the best performer regardless of sample size, group size ratio, effect size, and type of model and virtually always provides more accurate predictions. Moreover, CART, compared to the other prediction methods, provides the clinician with useful information regarding the relative importance of predictors in group separation with the advantage of producing human-readable rules^33^. Thus, we moved on to CART algorithm and found that IL-6 is the best predictor for COVID-19 disease. The addition of IL-8 well defined disease severity.

However, to obtain a best validation of the algorithm, we challenged the method with the determination of serum cytokines of patients enrolled in a different epidemic peak.

Characteristics of patients with COVID-19 have largely changed over time^3–5^. In Italy, patients who died in the second phase of the epidemic were older, more likely to be women, and had higher probability of superinfections, larger comorbidity burden, and longer survival from symptom onset compared to people who died in the first phase (March–May 2020)^5^. Here, we found that in wave 2 the cytokine storm profile developed at lower levels, compared to wave 1. Many cytokines that during wave 1 were increased in serum of COVID-19 patients were undistinguishable in patients compared to controls, during wave 2. For example, IL-4 and IL-5 levels were not increased in wave 2 COVID-19 patients, suggesting lack of type 2 immunity activation. Moreover, in comparison to wave 1, only IP-10 levels were significantly higher in severe versus mild COVID-19 patients, with no change of IL-1ra and IL-6 levels. IP-10 increase was not paralleled by IFN-γ increase, suggesting a direct relationship with viral pathogenic mechanisms.

The treatment approach has changed over the two periods, as critically ill patients in the second phase were less likely to receive antivirals and/or IL-6R inhibitors and more likely to be treated with steroids and FANS. Thus, the reduction of cytokine storm extent observed in wave 2 may reflect the different therapeutic strategies adopted in the two epidemic moments. For instance, in wave 2, the loss of IL-6 augmentation in severe COVID-19 may be explained with the treatment approach, definitely not based on tocilizumab administration.

The discrepancies we found among cytokine profiles in the two COVID-19 outbreaks has led to a modification of discriminative power of the previously identified algorithm. In particular, the challenge of the method with the results obtained during the second wave has revealed a different pattern of cytokines with best predictive performance and a reduction in the classification between mild and severe COVID-19. However, CART analysis was still able to define controls and mild COVID-19 patients, with high accuracy by an algorithm based on IL-6 concentration. Thereby, we have confirmed that IL-6 remains an excellent predictor and found that it represents a COVID-19 biomarker regardless the epidemic peak curves.

In conclusion, it is conceivable that a detailed knowledge of the role of single cytokines in SARS-CoV-2 infection and a prediction model built on cytokine levels might strongly help to foster novel diagnostic tools and to inform innovative therapeutic interventions.

## Supplementary Information

Supplementary Information.
