# Supplementary material for: Cytokine signature and COVID-19 prediction models in the two waves of pandemics
Source: Sci Rep. 2021 Oct 21;11:20793. doi: 10.1038/s41598-021-00190-0 (PMC8531346; doi:10.1038/s41598-021-00190-0)
Supplement: Supplementary file 1 — Supplementary Information. [file 41598_2021_190_MOESM1_ESM.pdf]

Cytokine signature and COVID-19 prediction models in the two waves of pandemics.

Serena Cabaro, Vittoria D'Esposito, Tiziana Di Matola, Silvia Sale, Michele Cennamo, Daniela Terracciano, Valentina Parisi, Giuseppe Portella, Francesco Beguinot, Luigi Atripaldi, Mario Sansone, Pietro Formisano.

Supplementary Tables

		TRUE		
		**Control**	**Mild COVID-19**	**Severe COVID-19**
LDA Predictions	**Control**	49	1	0
	**Mild**	0	43	1
	**Severe**	0	2	18
Accuracy	0.96, 95% CI : (0.91, 0.99)			
No Information Rate	0.42			
p-Value [Acc > NIR]	< 2.2e-16			
Kappa	0.944			

Supplementary Table 1: LDA Confusion Matrix and Statistics

	**Control**	**Mild COVID-19**	**Severe COVID-19**
Sensitivity	1.00	0.93	0.94
Specificity	0.98	0.98	0.97
Pos Pred Value	0.98	0.97	0.90
Neg Pred Value	1.00	0.95	0.98
Prevalence	0.42	0.40	0.16
Detection Rate	0.42	0.37	0.15
Detection Prevalence	0.43	0.38	0.17
Balanced Accuracy	0.99	0.96	0.96

Supplementary Table 2: LDA Statistics by Class

	LD1	LD2
PDGF	0.58	-0.81
IL-1b	3.91	1.70
IL-1ra	-0.18	1.93
IL-2	-6.49	11.01
IL-4	8.62	-1.04
IL-5	7.23	-1.18
IL-6	1.30	1.03
IL-7	1.03	-7.22
IL-8	0.39	-0.59
IL-9	4.97	-4.89
IL-10	6.15	0.53
IL-12	-4.16	0.77
IL-13	-2.39	-2.31
IL-15	0.25	-10.85
IL-17	-0.76	3.59
Eotaxin	-3.51	0.37
FGF-b	-6.47	-10.65
G-CSF	-2.22	2.49
GM-CSF	-4.59	5.76
IFN-g	-4.74	-3.20
IP-10	1.67	0.06
MCP-1	-0.14	-0.41
MIP-1a	2.02	-0.34
MIP-1b	-8.04	6.87
CCL5/RANTES	0.25	1.53
TNF-α	0.86	-6.36
VEGF	4.63	0.05

Supplementary Table 3: Coefficients for $\log_{10}$ cytokine concentrations to obtain LD1 and LD2 projections for LDA

	Control	Mild COVID-19	Severe COVID-19
PDGF	0.82	0.82	0.64
IL-1b	0.79	0.79	0.71
IL-1ra	0.89	0.75	0.89
IL-2	0.78	0.77	0.78
IL-4	0.88	0.88	0.79
IL-5	0.91	0.90	0.91
IL-6	0.99	0.94	0.99
IL-7	0.93	0.93	0.74
IL-8	0.96	0.91	0.96
IL-9	0.62	0.59	0.62
IL-10	0.97	0.97	0.96
IL-12	0.69	0.69	0.60
IL-13	0.63	0.65	0.65
IL-15	0.90	0.90	0.86
IL-17	0.74	0.73	0.74
Eotaxin	0.64	0.66	0.66
FGF-b	0.69	0.69	0.56
G-CSF	0.88	0.86	0.88
GM-CSF	0.70	0.70	0.69
IFN-g	0.89	0.89	0.85
IP-10	0.97	0.86	0.97
MCP-1	0.88	0.84	0.88
MIP-1a	0.92	0.88	0.92
MIP-1b	0.60	0.60	0.55
CCL5/RANTES	0.61	0.61	0.58
TNF-α	0.70	0.70	0.68
VEGF	0.89	0.89	0.88

Supplementary Table 4: AUC values of a group compared to the sum of the other two groups (i.e. CTRL versus Mild+Severe)

		TRUE		
		**Control**	**Mild COVID-19**	**Severe COVID-19**
CART Predictions	**Control**	44	2	0
	**Mild**	5	41	7
	**Severe**	0	3	12
Accuracy	0.85, 95% CI : (0.77, 0.91)			
No Information Rate	0.42			
p-Value [Acc > NIR]	< 2.2e-16			
Kappa	0.75			

Supplementary Table 5: CART Confusion Matrix and Statistics (Wave 1)

	Control	Mild COVID-19	Severe COVID-19
Sensitivity	0.89	0.89	0.63
Specificity	0.96	0.82	0.96
Pos Pred Value	0.95	0.77	0.80
Neg Pred Value	0.92	0.91	0.92
Prevalence	0.42	0.40	0.16
Detection Rate	0.38	0.35	0.10
Detection Prevalence	0.40	0.46	0.13
Balanced Accuracy	0.93	0.85	0.80

Supplementary Table 6: CART Statistics by Class (Wave 1)

		TRUE		
		**Control**	**Mild COVID-19**	**Severe COVID-19**
CART Predictions	**Control**	11	3	0
	**Mild**	4	23	9
	**Severe**	0	0	1
Accuracy	0.68, 95% CI : (0.54, 0.80)			
No Information Rate	0.50			
p-Value [Acc > NIR]	0.0081			
Kappa	0.43			

Supplementary Table 7: CART Confusion Matrix and Statistics (Challenge of CART - Wave 2 as test sample)

	Control	Mild COVID-19	Severe COVID-19
Sensitivity	0.73	0.88	0.10
Specificity	0.91	0.48	1.00
Pos Pred Value	0.78	0.63	1.00
Neg Pred Value	0.89	0.80	0.82
Prevalence	0.29	0.50	0.19
Detection Rate	0.21	0.45	0.01
Detection Prevalence	0.27	0.70	0.01
Balanced Accuracy	0.82	0.68	0.55

Supplementary Table 8: CART Statistics by Class (Challenge of CART - Wave 2 as test sample)

	AUC
IL-10	0.96
IL-6	0.93
IL-7	0.92
IL-8	0.91
IL-5	0.90
IL-15	0.89
VEGF	0.89
IFN-g	0.89
MIP-1a	0.88
IL-4	0.87
IP-10	0.86
G-CSF	0.85
MCP-1	0.83
PDGF	0.82
IL-1b	0.78
IL-2	0.76
IL-1ra	0.75
IL-17	0.72
GM-CSF	0.70
TNF-a	0.69
FGF-b	0.69
IL-12	0.68
Eotaxin	0.63
CCL5/RANTES	0.60
MIP-1b	0.59
IL-13	0.58
IL-9	0.51

Supplementary Table 9: AUC of ROC curve analysis of each cytokine in controls and mild COVID-19 patients (Wave 1).

		TRUE	
		**Control**	**Mild COVID-19**
CART Predictions	**Control**	44	2
	**Mild**	5	44
Accuracy	0.92, 95% CI : (0.85, 0.97)		
No Information Rate	0.51		
p-Value [Acc > NIR]	<2e-16		
Kappa	0.85		

Supplementary Table 10: CART Confusion Matrix and Statistics in controls and mild COVID-19 patients (Wave 1)

Sensitivity	0.95
Specificity	0.89
Pos Pred Value	0.89
Neg Pred Value	0.95
Prevalence	0.48
Detection Rate	0.46
Detection Prevalence	0.51
Balanced Accuracy	0.92

Supplementary Table11: CART Statistics in controls and mild COVID-19 patients (Wave 1)

		TRUE	
		**Control**	**Mild COVID-19**
CART Predictions	**Control**	11	3
	**Mild**	4	23
Accuracy	0.83, 95% CI : (0.68, 0.93)		
No Information Rate	0.63		
p-Value [Acc > NIR]	0.0055		
Kappa	0.63		

Supplementary Table 12: CART Confusion Matrix and Statistics in controls and mild COVID-19 patients (Challenge of CART - Wave 2 as test sample)

Sensitivity	0.88
Specificity	0.73
Pos Pred Value	0.85
Neg Pred Value	0.78
Prevalence	0.63
Detection Rate	0.56
Detection Prevalence	0.66
Balanced Accuracy	0.81

Supplementary Table 13: CART Statistics in controls and mild COVID-19 patients (Challenge of CART - Wave 2 as test sample)
